# Chloral hydrate-dependent reduction in the peptidoglycan-induced inflammatory macrophage response is associated with lower expression levels of toll-like receptor 2

**DOI:** 10.3892/etm.2014.1587

**Published:** 2014-02-26

**Authors:** QINGJUN PAN, YUAN LIU, XUEZHI ZHU, HUAFENG LIU

**Affiliations:** 1 Institute of Nephrology, Affiliated Hospital of Guangdong Medical College, Zhangjiang, Guangdong 524001, P.R. China; 2 Department of Paediatrics and Adolescent Medicine, Li Ka Shing Faculty of Medicine, University of Hong Kong, Hong Kong, SAR 999077, P.R. China; 3 Guangdong Yuehai Feed Group Co. Ltd., Zhangjiang, Guangdong 524001, P.R. China

**Keywords:** chloral hydrate, peptidoglycan, inflammatory response, macrophage, toll-like receptor 2

## Abstract

The aim of this study was to investigate the effect and mechanism of action of chloral hydrate on the peptidoglycan (PGN)-induced inflammatory macrophage response. The effect of chloral hydrate on the production of tumor necrosis factor α (TNF-α) and interleukin-6 (IL-6) by murine peritoneal macrophages with PGN-stimulation was investigated. In addition, RAW264.7 cells transfected with a nuclear factor-κB (NF-κB) luciferase reporter plasmid stimulated by PGN were used to study the effect of chloral hydrate on the levels NF-κB activity. Flow cytometry and western blotting were performed to investigate the expression levels of toll-like receptor 2 (TLR2) in the treated RAW264.7 cells. It was identified that chloral hydrate reduced the levels of IL-6 and TNF-α produced by the peritoneal macrophages stimulated with PGN. The levels of NF-κB activity of the RAW264.7 cells stimulated by PGN decreased following treatment with chloral hydrate, which was associated with a reduction in the expression levels of TLR2 and reduced levels of TLR2 signal transduction. These data demonstrate that chloral hydrate reduced the magnitude of the PGN-induced inflammatory macrophage response associated with lower expression levels of TLR2.

## Introduction

Chloral hydrate is a well-known sedative and anesthetic that is used in pediatric procedures, including echocardiograms and magnetic resonance imaging (1–5), and in animal experiments (6–9). Its safety and pharmacological mechanisms in clinical practice have been emphasized (10–12)

In a previous study, it was demonstrated that therapeutic concentrations of chloral hydrate delayed and reduced the magnitude of the inflammatory response and improved the survival rate of mice following the induction of liver injury with lipopolysaccharide and D-galactosamine. This altered inflammatory response was associated with the inhibitory effects of chloral hydrate on nuclear factor-κB (NF-κB) activity and the levels of serum proinflammatory cytokines induced by lipopolysaccharide (13).

Peptidoglycan (PGN) is a common and conserved component of the cell walls of Gram-positive (G+) bacteria, including *Staphylococcus aureus,* and has been used as a toll-like receptor 2 (TLR2)-specific ligand (14). It has been reported that PGN is detected in the blood of 80% of patients with serious bacterial infections (15). The mononuclear phagocyte system plays a crucial role against infection in the innate immune response, and murine macrophages are an important model for studying infection. Following stimulation with PGN, NK-κB becomes activated and macrophages release large quantities of the proinflammatory cytokines IL-6 and TNF-α. Simultaneously, the levels of TLR2 expression become upregulated (16,17). Activation of a murine macrophage cell line (RAW264.7) by PGN is mediated through the TLR2 signaling cascade, which involves the activation of a number of kinases, including p38 mitogen-associated protein kinase (MAPK), extracellular signal-regulated kinase (ERK) 1/2, inhibitor of NF-κB α (IκBα) and Akt (18,19).

In the present study, the effect of chloral hydrate on the production of the proinflammatory cytokines and the activity of NF-κB in PGN-stimulated murine peritoneal macrophages and RAW264.7, respectively, was investigated. The study includes an exploration of the mechanisms and an investigation of the effects of chloral hydrate treatment on the expression levels of TLR2 and TLR2 signal transduction in murine peritoneal macrophages and RAW264.7 cells stimulated with PGN.

## Materials and methods

### Reagents

PGN (*S. aureus*, strain DSM346), chloral hydrate (cat. no. 302-17-0) and trypsin-EDTA solution (10X; cat. no. T4174) were purchased from Sigma-Aldrich (St. Louis, MO, USA). Iscove’s modified Dulbecco’s medium (IMDM) and fetal bovine serum (FBS) were purchased from Gibco (Grand Island, NY, USA). The TNF-α and IL-6 ELISA kits were obtained from R&D Systems, Inc. (Minneapolis, MN, USA). The fluorescein isothiocyanate (FITC)-conjugated anti-mouse TLR2 antibody (clone TL2.5), FITC-conjugated mouse IgG1 (isotype control), anti-mouse TLR2 antibody (clone TL2.5), horseradish peroxidase (HRP)-conjugated goat anti-mouse secondary antibody and Cell Staining Buffer were purchased from BioLegend, Inc. (San Diego, CA, USA). The antibodies against p38 MAPK, phospho-p38 MAPK, ERK 1/2, phospho-ERK1/2, IκBα, phospho-IκBα, Akt, phospho-Akt and β-actin were obtained from Cell Signaling Technology, Inc. (Waltham, MA, USA). The NF-κB-luciferase and β-galactosidase reporter vectors and the dual luciferase reporter assay system were obtained from Promega Corporation (Madison, WI, USA). The Immobilon-P membrane was obtained from Millipore UK Ltd. (Watford, UK) and the enhanced chemiluminescence (ECL) kit was purchased from Amersham Life Science Ltd. (Little Chalfont, UK).

### Effects of chloral hydrate on proinflammatory cytokine production by murine peritoneal macrophages stimulated with PGN

The separation and cultivation of mouse peritoneal macrophages were performed as previously described (20). The Institutional Review Board of the Affiliated Hospital of Guangdong Medical College (Zhangjiang, China) approved the removal of the macrophages from the six-week-old BALB/c mice were purchased from Experimental Animal Center of Southern Medical University in the present study. The cells were seeded at a density of 1×10^5^ cells/well in 12-well plates in IMDM supplemented with 5% heat-inactivated FBS in a humidified 5% CO_2_ and air incubator at 37°C. The supernatants were collected at 6, 12 and 24 h following stimulation with PGN (1 μg/ml) or PGN (1 μg/ml) plus chloral hydrate (0.25 and 1 mg/ml) and saline-treated controls. The supernatants were then stored at −80°C prior to the measurement of the levels of TNF-α and IL-6 using the corresponding ELISA kits according to the manufacturer’s instructions.

### Effects of chloral hydrate on the levels NF-κB activity in PGN-stimulated RAW264.7 cells transfected with a NF-κB luciferase reporter plasmid

The RAW264.7 cells were purchased from the Type Culture Collection of the Chinese Academy of Sciences (Shanghai, China) and cultured in IMDM (5% FBS) and maintained at 37°C in 5% CO_2_. The cells were seeded in 25-mm dishes at a density of 1×10^6^ cells per well. These cells were harvested with 0.25 g/l trypsin-EDTA 48 h later, and 5×10^6^ RAW264.7 cells were transiently transfected with NF-κB-luciferase (5 μg) and β-galactosidase reporter (5 μg) vectors (for normalization of the efficiency of the transfection) in a volume of 400 μl by electroporation at 250 V, 960 μF capacitance pulse as previously described (21). The cells were subsequently washed once in IMDM and then split into 50 wells (100 μl/well) and cultured for 24 h in IMDM (5% FBS), prior to stimulation with PGN (1 μg/ml) for 6 or 12 h in the presence or absence of chloral hydrate (0.25 or 1 mg/ml). For the luciferase activity assays, the transfected RAW264.7 cells were stimulated for 6 or 12 h and subsequently harvested and lysed, and the luciferase activity in the extracts was assayed with the dual luciferase reporter assay system according to the manufacturer’s instructions. The luciferase activity of the cell extracts is expressed as the fold of luciferase-induction over that of a saline-treated control.

### Effects of chloral hydrate on PGN-induced upregulation of TLR2 expression levels in RAW264.7 cells

Flow cytometry was performed to investigate the levels of TLR2 expression in the RAW264.7 cells. The RAW 264.7 cells were cultured in IMDM (5% FBS) for 12 h with PGN (1 μg/ml) in the presence or absence of chloral hydrate (0.25 mg/ml). After harvesting, the cells were incubated with FITC-conjugated anti-mouse-TLR2 (1 μg) for 30 minutes at room temperature without permeabilization. As the isotype control, FITC-conjugated mouse IgG1 was used to detect nonspecific staining. The cells were washed twice with Cell Staining Buffer. A six-parameter flow cytometer (FACScan; BD Biosciences, San Jose, CA, USA) was used in the data acquisition. The analysis of the acquired data was performed using CellQuest software (BD Biosciences Immunocytometry Systems, San Jose, CA, USA). The mean channel fluorescence intensity (MFI) derived from the fluorescence histogram was used to study the levels of TLR2 expression. The MFI was calculated as a ratio and recorded as the MFI of the TLR2 antibody divided by the MFI of a normal (saline) control.

The TLR2 expression levels in the extracts from the RAW264.7 cells stimulated with PGN (1 μg/ml) for 12 h in the presence or absence of chloral hydrate (0.25 mg/ml) were semi-quantitatively analyzed by western blotting using the anti-mouse TLR2 antibody (clone TL2.5). The extracts from the RAW264.7 cells were analyzed by sodium dodecyl sulfate-polyacrylamide gel electrophoresis. The immunoblotting was performed by a standard western blotting procedure onto the Immobilon membrane. HRP-conjugated goat anti-mouse secondary antibody followed by ECL was used for the detection of the TLR2 signal. The semi-quantitative analysis of the extracts from the RAW264.7 cells was conducted using BandScan software, version 5.0 (Glyko Inc., Novato, CA, USA).

### Effects of chloral hydrate on PGN-induced TLR2 signal transduction in RAW264.7 cells

The TLR2 expression levels in the extracts from the RAW264.7 cells stimulated with PGN (1 μg/ml) for 12 h in the presence or absence of chloral hydrate (0.25 mg/ml) were semi-quantitatively analyzed by western blotting using antibodies against MAPK, phospho-p38 MAPK, ERK 1/2, phospho-ERK1/2, IκBα, phospho-IκBα, Akt, phospho-Akt and β-actin. Western blot analysis and detection were performed as previously described in this study. The semi-quantitative analysis of the extracts from the RAW264.7 cells was conducted using BandScan software.

### Statistical analysis

The data are presented as the mean ± standard deviation, and the statistical analysis was performed with SPSS statistical software, version 15.0 (SPSS, Inc., Chicago, IL, USA). The statistical significance between the two groups was determined by the unpaired Student’s t-test. A value of P<0.05 was considered to indicate a statistically significant difference.

## Results

### Chloral hydrate treatment reduces the levels of IL-6 and TNF-α produced by PGN-stimulated peritoneal macrophages

**Figure 1:**
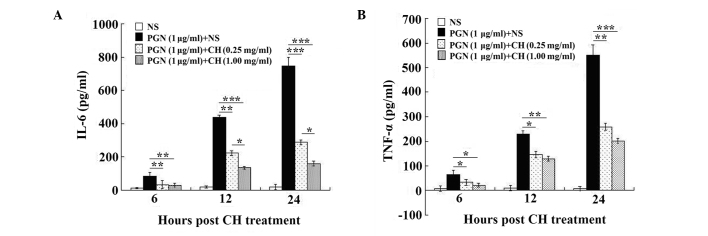
Chloral hydrate (CH) treatment decreased the levels of IL-6 and TNF-α production by peritoneal macrophages following PGN stimulation. The effects of chloral hydrate (0.25 and 1 mg/ml) on the levels of (A) IL-6 and (B) TNF-α produced by peritoneal macrophages (1×10^6^ cell/well) following PGN stimulation for 6, 12 and 24 h. The data are expressed as the mean ± SD of three independent experiments. ^*^P<0.05, ^**^P<0.01 and ^***^P<0.001. IL-6, interleukin-6; TNF-α, tumor necrosis factor α; NS, normal saline; PGN, peptidoglycan.

The results showed that the levels of IL-6 (Fig. 1A) and TNF-α (Fig. 1B) production increased sharply post-stimulation with PGN (1 μg/ml) for 6, 12 and 24 h, compared with those of the untreated cells, and decreased significantly following the chloral hydrate treatment (0.25 and 1 mg/ml), compared with those of the cells treated with PGN alone (all P<0.01). In addition, the higher concentration of chloral hydrate (1 mg/ml) significantly reduced the levels of IL-6 production following stimulation with PGN (1 μg/ml) for 12 and 24 h compared with those of the cells treated with the lower concentration of chloral hydrate (0.25 mg/ml; all P<0.05; Fig. 1A).

### Chloral hydrate-treatment reduces the levels of NF-κB activity in PGN-stimulated RAW264.7 cells

**Figure 2:**
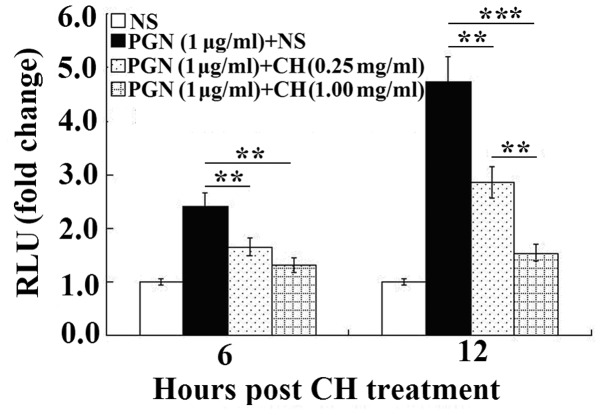
Chloral hydrate (CH) treatment decreased the levels of NF-κB activity of the RAW264.7 cells during PGN stimulation. One day after the RAW264.7 cells were co-transfected with NF-κB-luciferase- and β-galactosidase-reporter vectors by electroporation, PGN (1 μg/ml) or PGN (1 μg/ml) plus chloral hydrate (0.25 or 1 mg/ml) was added to the medium and incubated for 6 or 12 h. The luciferase activity of the cell extracts was measured and expressed as the fold of luciferase-induction over that of a saline-treated control ± SD of three independent experiments. ^**^P<0.01 and ^***^P<0.001. RLU, relative light unit; PGN, peptidoglycan; NF-κB, nuclear factor-κB.

The levels of luciferase activities in the transfected RAW264.7 cells were measured at 6 and 12 h after PGN stimulation (Fig. 2). The results are presented as a fold of induction over the values obtained in the normal saline groups. As shown in Fig. 2, ~1.5- and 3.5-fold increases in the luciferase activity were observed following the PGN stimulation for 6 h and 12 h, respectively, compared with those of the untreated cells. The effect of PGN-stimulation was significantly reduced by the chloral hydrate treatment (0.25 and 1 mg/ml; all P<0.01). A higher concentration of chloral hydrate (1 mg/ml) led to a significantly larger reduction in the levels of luciferase activity after 12 h than did the lower concentration of chloral hydrate (0.25 mg/ml; P<0.01).

### Chloral hydrate-treatment reduces the increased TLR2 expression levels in PGN-treated RAW264.7 cells

The two concentrations (0.25 and 1 mg/ml) of chloral hydrate treatment were shown to significantly reduce the inflammatory response of the peritoneal macrophages and RAW264.7 cells following PGN-stimulation compared with that of the macrophages and RAW264.7 cells treated with PGN alone, and the effect of chloral hydrate treatment on the expression levels of the receptor for PGN, TLR2, was tested using the lowest effective concentration of chloral hydrate (0.25 mg/ml) in the RAW264.7 cells.

**Figure 3:**
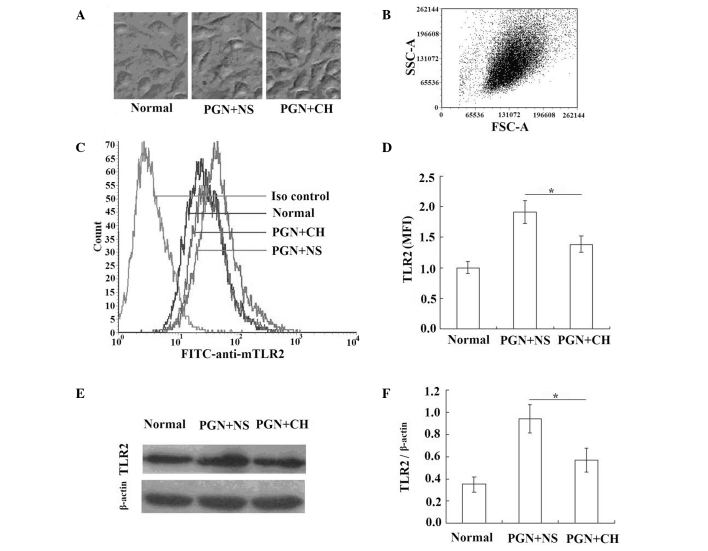
Chloral hydrate (CH) treatment reduced the expression levels of TLR2 in the PGN-stimulated RAW264.7 cells. (A) The RAW 264.7 cells were cultured in IMDM supplemented with 5% heat-inactivated FBS for 12 h with PGN (1 μg/ml) in the presence or absence of chloral hydrate (0.25 mg/ml). Flow cytometry was performed to investigate the expression levels of TLR2 in the treated RAW264.7 cells, presented as (B) dot plots and (C) histograms. The MFI derived from the fluorescence histogram was used to study the levels of the cell surface TLR2 expression. (D) The MFI was calculated as a ratio and recorded as the MFI of the TLR2 antibody divided by the MFI of the normal control (normal saline group). The semi-quantitative analysis of the extracts from the RAW264.7 cells by BandScan and the ratio of TLR2 to β-actin is shown; (E) shows a western blot of β-actin and TLR2 in the normal, PGN+NS and PGN+CH groups and (F) shows a quantitation of them. The data are expressed as the mean ± SD of three independent experiments. ^*^P<0.05. PGN, peptidoglycan; FITC, fluorescein isothiocyanate; TLR2, toll-like receptor 2; IMDM, Iscove’s modified Dulbecco’s medium; MFI, mean channel fluorescence intensity.

The cells were cultured in the IMDM supplemented with 5% heat-inactivated FBS for 12 h with or without PGN (1 μg/ml) in the presence or absence of chloral hydrate (0.25 mg/ml; Fig. 3A) and harvested, and the levels of the MFI of TLR2 were measured. In the RAW264.7 cells stimulated by PGN (1 μg/ml) for 12 h, the TLR2 expression levels were significantly increased (Fig. 3B–D) compared with those in the unstimulated cells, and the chloral hydrate treatment (0.25 mg/ml) significantly reduced the upregulation of the levels of TLR2 stimulated by PGN (P<0.05; Figs. 3B–D).

The corresponding semi-quantitative analysis of the levels of TLR2 expression in the extracts of the RAW264.7 cells by western blotting showed a similar trend (Fig. 3E and F). This shows that if the amounts of TLR2 signal are considered in relation to the signal of the household protein β-actin, the increase and the reduction of the TLR2 signals are not caused by cell death or variations in the number of cells in the sample.

### Treatment of PGN-induced RAW264.7 cells with chloral hydrate reduces the levels of TLR2 signal transduction

Although chloral hydrate-treatment reduced the increased TLR2 expression levels in the PGN-treated RAW264.7 cells, whether the levels of TLR2-associated signal transduction molecules, including phospho-p38 MAPK, phospho-ERK1/2, phospho-IκBα and phospho-Akt (18,19), were reduced was unknown.

The effect of chloral hydrate on TLR2 signal transduction was investigated by western blotting, and the lower effective concentration (0.25 mg/ml) was selected to test the effect of chloral hydrate treatment on TLR2 signal transduction in RAW264.7 cells.

**Figure 4:**
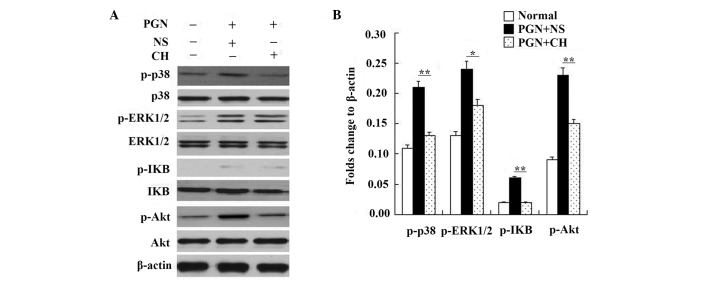
RAW264.7 cells were treated with PGN (1 μg/ml) for 12 h in the presence or absence of chloral hydrate (CH; 0.25 mg/ml). The cell lysates were (A) immunoblotted with antibodies against MAPK, phospho-p38 MAPK, ERK 1/2, phospho-ERK1/2, IκBα, phospho-IκBα, Akt, phospho-Akt or β-actin and (B) semi-quantified to show the reduction in signal. The experiments were repeated three times with similar results. PGN, peptidoglycan; ERK, extracellular signal-regulated kinase; MAPK, mitogen-associated protein kinase.

Following culture in IMDM supplemented with 5% heat-inactivated FBS for 12 h with or without PGN (1 μg/ml) in the presence or absence of chloral hydrate (0.25 mg/ml), the upregulation of signal transduction of TLR2 in RAW264.7 stimulated by PGN (1 μg/ml) for 12 h was tested for significance with western blotting. The upregulation of TLR2 signal transduction stimulated by PGN was reduced following chloral hydrate (0.25 mg/ml) treatment (Fig 4).

## Discussion

The mechanisms of bacteria-induced acute inflammation and anti-cytokines as therapeutic agents have been reevaluated. The majority of the reported studies on sepsis-associated anti-cytokine therapies have been unpromising or disappointing (22–24). Intensive investigative efforts have been made with the aim of developing novel drugs and treatment strategies for severe sepsis and acute inflammation.

The process for the development and approval of novel drugs in therapeutic programs requires a large amount of time and effort. Novel uses for traditional, old drugs may accelerate the development and application of new therapies. For example, the bisphosphonate zoledronic acid, which has been used to treat osteoporosis and similar diseases, also decreases breast cancer metastasis (25), and thalidomide, which was originally used to alleviate nausea and morning sickness, is able to treat multiple myeloma (26). The identification of the protective effects of certain anesthetics and sedatives against infection or inflammation (27–30) may yield novel insights into anti-inflammatory therapies.

As a traditional anesthetic, chloral hydrate is typically used in patients and animal models and may be administered by mouth, injection and direct placement into the intestine/small bowel. Chloral hydrate is unlike isoflurane, an inhalation agent that must be inhaled continuously and is expensive. Chloral hydrate is able to be used freely in a number of countries, but other sedatives, for example ketamine, are under strictly regulated conditions in China and are not approved for patients under 16 years old by the US Food and Drug Administration (1).

The present study in murine peritoneal macrophages showed that chloral hydrate treatment reduced the rise of the inflammatory cytokine levels induced by PGN stimulation (Fig. 1), indicating that the effect of chloral hydrate on inflammation may be attributed to the inhibition of the macrophage function. The TNF-α and IL-6 levels sharply increased at 6, 12 and 24 h after the PGN-challenge. Similar studies using PGN (10 or 25 μg/ml) have been performed by Shirasawa *et al* (31) and Wang *et al* (32), although they tested the levels at 24 h after one challenge. The treatments with different concentrations (0.25 and 1.0 mg/ml) of chloral hydrate significantly reduced the rise of inflammatory cytokine levels at the indicated time points compared with those of the cells treated with PGN alone (Fig. 1).

In the present study, whether chloral hydrate regulates PGN-induced NF-κB-dependent gene transcription was investigated with a NF-κB-dependent luciferase reporter assay in RAW264.7 cells co-transfected with a β-galactosidase control plasmid. The results revealed that chloral hydrate treatment reduces the levels of NF-κB activity (Fig. 2). As NF-κB plays a key role in the transcriptional regulation of proinflammatory cytokine expression, this result suggests that chloral hydrate may affect cytokine expression by influencing the activity of NF-κB. After 6 and 12 h of PGN challenge, the respective increases of the levels of NF-κB-induced luciferase activity in the RAW264.7 cells were ~1.5- and 3.5-fold compared with the basal level. A similar study using PGN (5 μg/ml) was performed by Ito *et al* (33), and they observed the same effect.

The present also study examined whether chloral hydrate modulates the PGN-induced upregulation of TLR2 expression levels. Flow cytometry was used to analyze the effect of chloral hydrate on the expression levels of TLR2 in RAW264.7 cells stimulated by PGN. The expression levels of TLR2 in the RAW264.7 cells were significantly upregulated following PGN stimulation compared with those in the untreated cells. Similar results have been demonstrated by Chen *et al* (16). Following the chloral hydrate treatment, the marked upregulation of the TLR2 expression levels in response to PGN exposure was significantly reduced (Fig. 3), which is consistent with the effect of chloral hydrate on the levels of NF-κB activity and inflammatory cytokine production by RAW264.7 cells stimulated with PGN.

Having identified that the clear upregulation of TLR2 expression levels in response to PGN exposure was markedly diminished following treatment with chloral hydrate, the present study evaluated the effects of PGN and chloral hydrate on TLR2 signal transduction by analyzing the levels of signaling species, including p38 MAPK, ERK1/2, IκBα and Akt. In the RAW264.7 cells, PGN markedly induced the activation by phosphorylation of p38 MAPK, ERK1/2, IκBα and Akt, but this phosphorylation was reduced by the chloral hydrate treatment (Fig. 4).

The present study showed, to the best of our knowledge, for the first time that chloral hydrate reduced the PGN-induced upregulation of the levels of NF-κB activity and TNF-α and IL-6 production by macrophages in a time- and concentration-dependent manner. It was demonstrated that this reduction was associated with attenuation of the upregulation of PGN-induced-TLR2 expression and TLR2 signal transduction levels. Knowing the mechanisms of chloral hydrate treatment in inflammation provides opportunities to design novel therapeutic strategies for reducing the inflammation caused by G+ organisms.
